# Diagnosis of ulnar nerve entrapment anterior to the medial epicondyle by ultrasound elastography and diffusion tensor imaging with fiber tractography: a case report

**DOI:** 10.1007/s00276-021-02881-9

**Published:** 2022-01-13

**Authors:** Guillaume Jaques, Fabio Becce, Jean-Baptiste Ledoux, Sébastien Durand

**Affiliations:** 1 Department of Plastic and Hand Surgery, Lausanne University Hospital and University of Lausanne, 1011 Lausanne, Switzerland; 2 Department of Diagnostic and Interventional Radiology, Lausanne University Hospital and University of Lausanne, 1011 Lausanne, Switzerland

**Keywords:** Diffusion tensor imaging, Tractography, Elastography, Ulnar nerve, Cubital tunnel syndrome, Nerve surgery

## Abstract

Ulnar/cubital tunnel syndrome is the second most common compressive neuropathy of the upper limb. Permanent location of the ulnar nerve anterior to the medial epicondyle is extremely rare, with only five cases reported in the literature. Using ultrasound elastography and diffusion tensor imaging with fiber tractography, we diagnosed a case in which ulnar nerve entrapment was associated with anterior nerve location. Surgical release confirmed the diagnosis and the patient was symptom free 3 months after surgery.

**Supplementary Information:**

The online version contains supplementary material available at 10.1007/s00276-021-02881-9.

## Introduction

Ulnar/cubital tunnel syndrome is the second most common compressive neuropathy of the upper limb with an estimated incidence of 25 cases per 100,000 individuals [12]. Its etiology is mostly idiopathic, although this neuropathy is favored by repetitive elbow flexion, repetitive trauma, and disproportionate strain [10]. The ulnar nerve runs posterior to the medial epicondyle in most fetuses [11] and adults. Transient ulnar nerve dislocation anterior to the medial epicondyle and ulnar nerve subluxation are common in ulnar nerve instability [6]. Apart from nerve transposition surgery, congenital, permanent anterior location of the ulnar nerve is extremely rare, with only five cases reported in the literature since 1980 (Table 1) [12]. Snapping triceps [7], elbow fracture [4], and trisomy 13 [1] have all been described in association with anterior location of the ulnar nerve. Here, we report a rare case of ulnar nerve entrapment anterior to the medial epicondyle using advanced imaging techniques.

**Table 1:** Case reports on permanent ulnar nerve location anterior to the medial epicondyle

First author/year	Number of cases	Age (sex)	Side	History/symptoms
Aziz (1980) [1]	1	Neonate (female)	Bilateral	Trisomy 13 (dissection)
Davis (2006) [2]	1	43 years (female)	Left	Previous elbow joint fracture
				Pain, numbness, weakness of the left hand
Satteson (2015) [8]	2	66 years (male)	Left	Pain, tingling of the ring and little finger
				Previous mid-forearm fracture
		21 years (female)	Right	Numbness, tingling of the ring and little finger
Imao (2020) [3]	1	43 years (female)	Right	Painful snapping of the medial head of the triceps
Jaques (2021) (current report)	1	56 years (male)	Left	Pain, tingling of the ring and little finger

## Case report

A 56-year-old man, without history of trauma or surgery of the upper limb, reported numbness and tingling in the little finger and ulnar aspect of the ring finger of the left hand. Sensory examination revealed a normal two-point discrimination (4 mm) according to the American Society for Surgery of the Hand. Froment’s sign and Wartenberg’s sign were both absent and Tinel’s sign was positive proximal to the cubital tunnel at the elbow. The patient was graded 1 according to McGowan’s grading system. Electroneuromyography results showed a significant decrease in both ulnar nerve conduction velocity and amplitude of the nerve potential in the cubital tunnel.

**Fig. 1:**
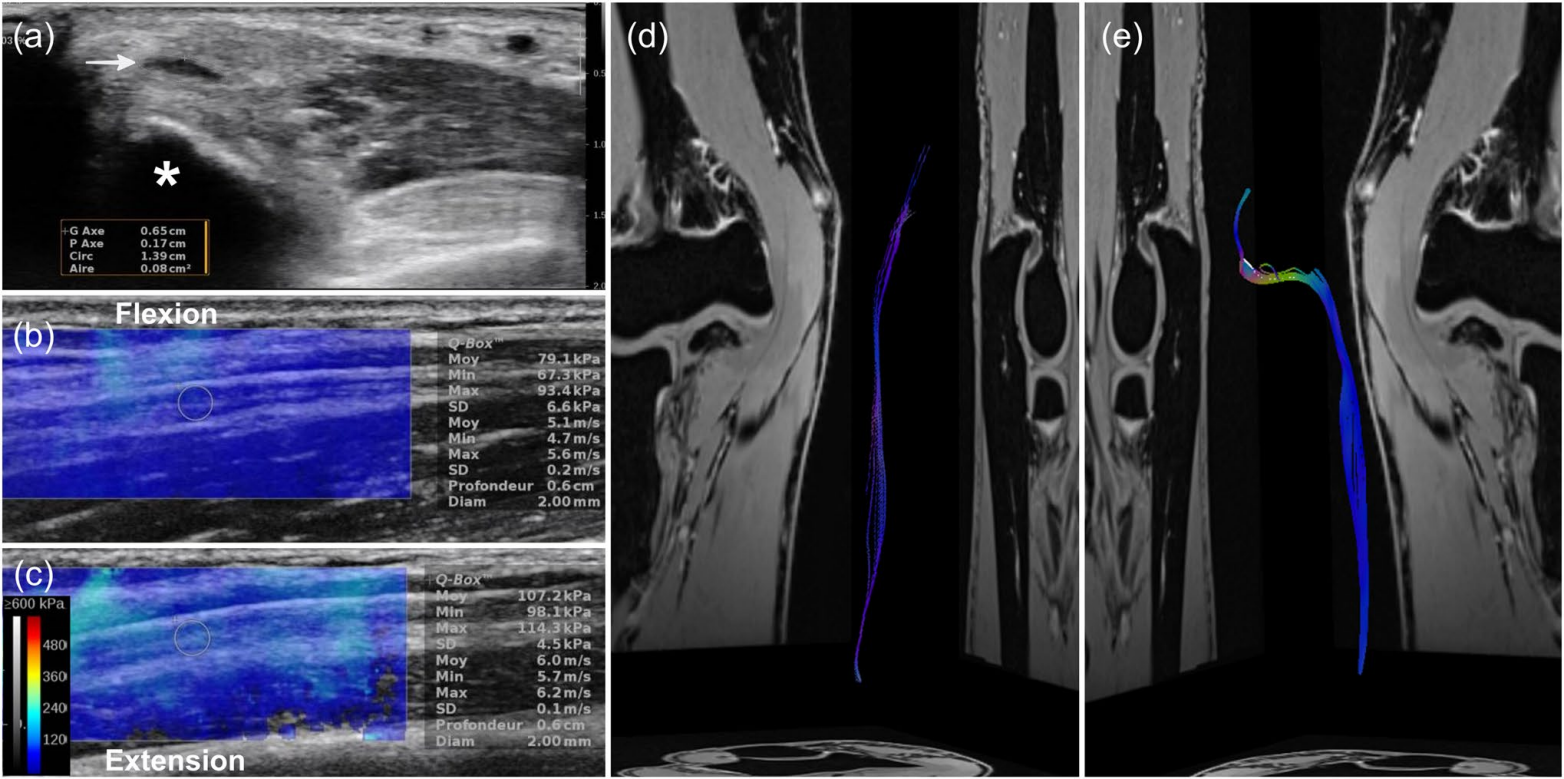
Conventional ultrasound and SWE, and DTI with fiber tractography. **a** Axial/transverse B-mode ultrasound image of the left ulnar nerve with CSA measurement at the compression site (0.08 cm^2^, white arrow). The ulnar nerve is located anterior to the medial epicondyle (asterisk). **b** SWE image and measurement of the left ulnar nerve in complete elbow flexion (mean, 79.1 kPa; SD, 6.6 kPa). **c** SWE image and measurement of the left ulnar nerve in complete elbow extension (mean, 107.2 kPa; SD, 4.5 kPa). DTI fiber tractography reconstructions of the right and left ulnar nerves. **d** Right elbow showing the normal pathway of the right ulnar nerve located posterior to the medial epicondyle within the cubital tunnel. **e** Abnormal “S-shape” pathway of the left ulnar nerve running anterior to the medial epicondyle outside the cubital tunnel

Ultrasound was performed using an Aixplorer™ system (SuperSonic Imagine, Aix-en-Provence, France) with a high-resolution 5–18 MHz linear array transducer (SuperLinear™ SL18-5; SuperSonic Imagine). In B-mode, we observed a permanent anterior location of the ulnar nerve relative to the medial epicondyle at the left elbow (Fig. 1a). There were no signs of nerve instability, triceps muscle abnormality, or triceps snapping syndrome. On the contralateral (right) elbow, the ulnar nerve ran normally posterior to the medial epicondyle. The CSA of the left ulnar nerve (0.16 cm^2^) was increased proximal to the cubital tunnel, with distal nerve flattening (0.08 cm^2^) at the level of the medial epicondyle. Shear-wave elastography (SWE) of the ulnar nerve using a 3-mm^2^ Q-Box (quantitative box) focus area was performed at the distal part of the arm in the longitudinal (long axis) plane. The shear elastic modulus of the left ulnar nerve increased from 79.1 to 107.2 kPa with elbow extension (Fig. 1b, c). On the normal contralateral side, the shear elastic modulus of the right ulnar nerve increased from 108.6 to 40.2 kPa with elbow extension.

MRI was performed on a 3-T system (MAGNETOM Prisma^fit^; Siemens Healthineers, Erlangen, Germany) with a transmit–receive knee coil. DTI was acquired using the following parameters: 37 sections; section thickness, 4 mm; intersection gap, 0.8 mm; repetition time, 5400 ms; echo time, 60 ms; fat suppression with spectral attenuated inversion recovery; reconstructed voxel size after interpolation, 1.00 × 1.00 × 4.00 mm^3^; *b* values, 0–700 s/mm^2^; parallel imaging (GRAPPA) acceleration factor, 2; and total scan time, 6 min 34 s. Fiber tractography reconstructions were obtained using a dedicated post-processing software (Numaris X; Siemens Healthineers). We observed a slight numerical difference in the fractional anisotropy values of the ulnar nerve at the level of the cubital tunnel between the left pathological elbow (mean 0.36; SD 0.07) and right normal side (mean 0.42; SD 0.06). Ulnar nerve tractography showed asymmetric fiber orientation of the left and right ulnar nerves (Fig. 1d, e; Supplementary Fig. 1), with an “S-shape” aberrant anterior pathway of the left ulnar nerve relative to the medial epicondyle (Fig. 1e; Supplementary Fig. 1).

**Fig. 2:**
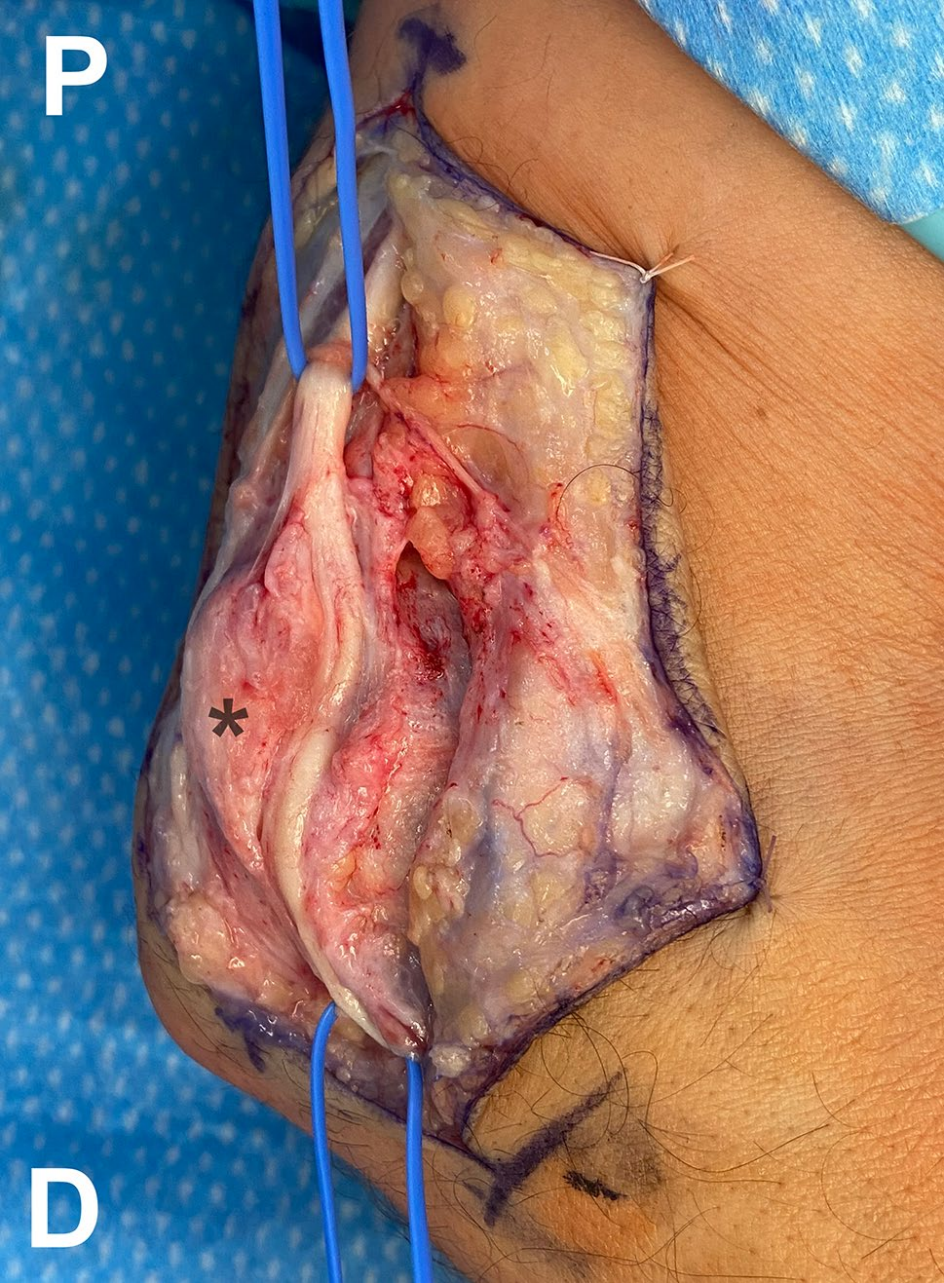
Intraoperative photograph of the medial aspect of the left elbow showing complete surgical release of the left ulnar nerve located anterior to the medial epicondyle (asterisk). *P* proximal; *D* distal

Surgical release of the left ulnar nerve was performed under axillary nerve block and tourniquet. A 12–15-cm longitudinal incision was performed posterior to the medial epicondyle. The ulnar nerve was located anterior to the medial epicondyle (Fig. 2) and complete release of all nerve compression points was performed: the aponeurosis of the flexor carpi ulnaris, the tunnel outlet between the two muscle heads of the flexor carpi ulnaris, the intermuscular septum, and the superficial fibers of the conjoined tendon of the flexor-pronator muscle mass. No instability of the ulnar nerve was observed after anterior release. The patient was symptom free with complete functional recovery at 3 months after surgery.

## Discussion

Ulnar neuropathy is classically assessed by clinical history, physical examination, and electroneuromyography, the latter representing a key diagnostic tool [12]. However, electroneuromyography also has some disadvantages as it is time consuming, can be painful, and has a limited sensitivity. High-resolution ultrasound imaging has gained attention recently as a complementary diagnostic test for peripheral nerve injuries [9]. Conventional ultrasound can provide information about the anatomical site of the lesion and changes in morphology and echogenicity of the nerve: loss of the nerve’s normal fascicular architecture, and increased/decreased nerve CSA or echogenicity in B-mode; increased nerve vascularity in color Doppler; and abnormal anatomical structures (e.g., accessory muscle) causing nerve injury [9]. Dynamic ultrasound examination may also demonstrate reduced/excessive nerve mobility with instability/transient subluxation over the medial epicondyle. Previous studies reported that patients with ulnar neuropathy at the elbow have an increased CSA compared to healthy controls, but the cutoff value above which nerve CSA is considered abnormal varies widely among authors [8]. SWE is a recently developed ultrasound technique that provides quantitative values of soft-tissue stiffness, including muscles, tendons, and nerves. It can help improve the diagnostic performance of ultrasound for various neuropathies involving the median, sciatic, tibial, and ulnar nerves [9, 10]. SWE has the potential to serve as an additional non-invasive test for assessing nerve tension by estimating its mechanical properties, such as stiffness.

In our case, B-mode ultrasound features suggested the diagnosis of ulnar nerve compression anterior to the medial epicondyle. SWE confirmed the anterior location of the ulnar nerve relative to the rotation axis of the elbow, with a decreased tension in the ulnar nerve during elbow flexion: the values of the shear elastic modulus in the ulnar nerve decreased from 107.2 to 79.1 kPa with elbow flexion. These shear elastic modulus values were comparable to those recently reported in patients before and after anterior transposition of the ulnar nerve [5]. Due to the absence of extrinsic compression factors in this anterior nerve location, excessive friction and stretching are other potential mechanisms to consider.

The diagnosis of ulnar neuropathy with conventional MRI sequences is challenging because of the significant overlap with findings in asymptomatic individuals [6]. An increased T2 signal in the ulnar nerve may suggest ulnar nerve entrapment, while an increased nerve CSA may distinguish severe from mild injuries [2]; however, the accuracy and reliability of these findings remain debated. Diffusion-weighted MRI can provide a measure of the motion of water molecules in tissues [3]. When diffusion-sensitizing gradients are applied from multiple directions (at least six), the diffusion of water in a voxel can be modeled as an ellipsoid by estimating the diffusion tensor, which is the basic principle of DTI. Diffusion tensor tractography refers to the analysis and reconstruction of data obtained by DTI, by which the orientation of nerve fibers can be determined to trace specific neural pathways. Using dedicated post-processing algorithms, fiber tractography images can be generated by linking the direction of maximum diffusivity in adjacent voxels. Tractography can demonstrate axon bundles and thereby depict the nerve integrity [13]. Assessment by DTI also provides new information about the effects of the disease process on peripheral nerve tissue microstructure in chronic inflammatory demyelinating polyradiculoneuropathy, permits the identification of normal nerve tissue prior to nerve tumor surgery, and can help identify pathological changes of the myelin sheath in peripheral nerve entrapments.

In our case, DTI combined with fiber tractography provided not only valuable three-dimensional anatomical information for the preoperative planning by precisely depicting the aberrant pathway of the pathological ulnar nerve relative to the medial epicondyle and the cubital tunnel but also the location and severity of the nerve compression, compared to the normal contralateral ulnar nerve.

## Conclusion

It is of utmost importance that peripheral nerve surgeons are aware of the existence of this rare entity (i.e., congenital, permanent anterior location of the ulnar nerve relative to the medial epicondyle) in patients with cubital nerve syndrome due to potential iatrogenic injuries during surgery. We recommend that preoperative ultrasound becomes a routine diagnostic test for patients with symptoms of ulnar nerve entrapment at the elbow. While fine anatomical details can be obtained from high-resolution three-dimensional MRI sequences, advanced quantitative imaging, such as SWE and DTI, with fiber tractography can help determine the presence, location, and severity of peripheral nerve injuries, fiber tractography further allowing three-dimensional visualization of the peripheral nerve pathways.

## Supplementary Information

Below is the link to the electronic supplementary material.

Supplementary file 1: Supplementary Fig. 1. DTI fiber tractography reconstructions of the right and left ulnar nerves overlaid with axial/transverse morphological MRI sequences. **a** On the right, the ulnar nerve runs posterior to the medial epicondyle within the cubital tunnel, **b** while on the left the nerve is abnormally located anterior to the medial epicondyle. (TIFF 6680 KB)

## Data Availability

Not applicable.

## Abbreviations

CSA: Cross-sectional area
DTI: Diffusion tensor imaging
MRI: Magnetic resonance imaging
SWE: Shear-wave elastography
SD: Standard deviation
