# Supplementary figures and images for: Diagnosis of ulnar nerve entrapment anterior to the medial epicondyle by ultrasound elastography and diffusion tensor imaging with fiber tractography: a case report
Source: Surg Radiol Anat. 2022 Jan 13;44(2):201–5. doi: 10.1007/s00276-021-02881-9 (PMC8831343; doi:10.1007/s00276-021-02881-9)

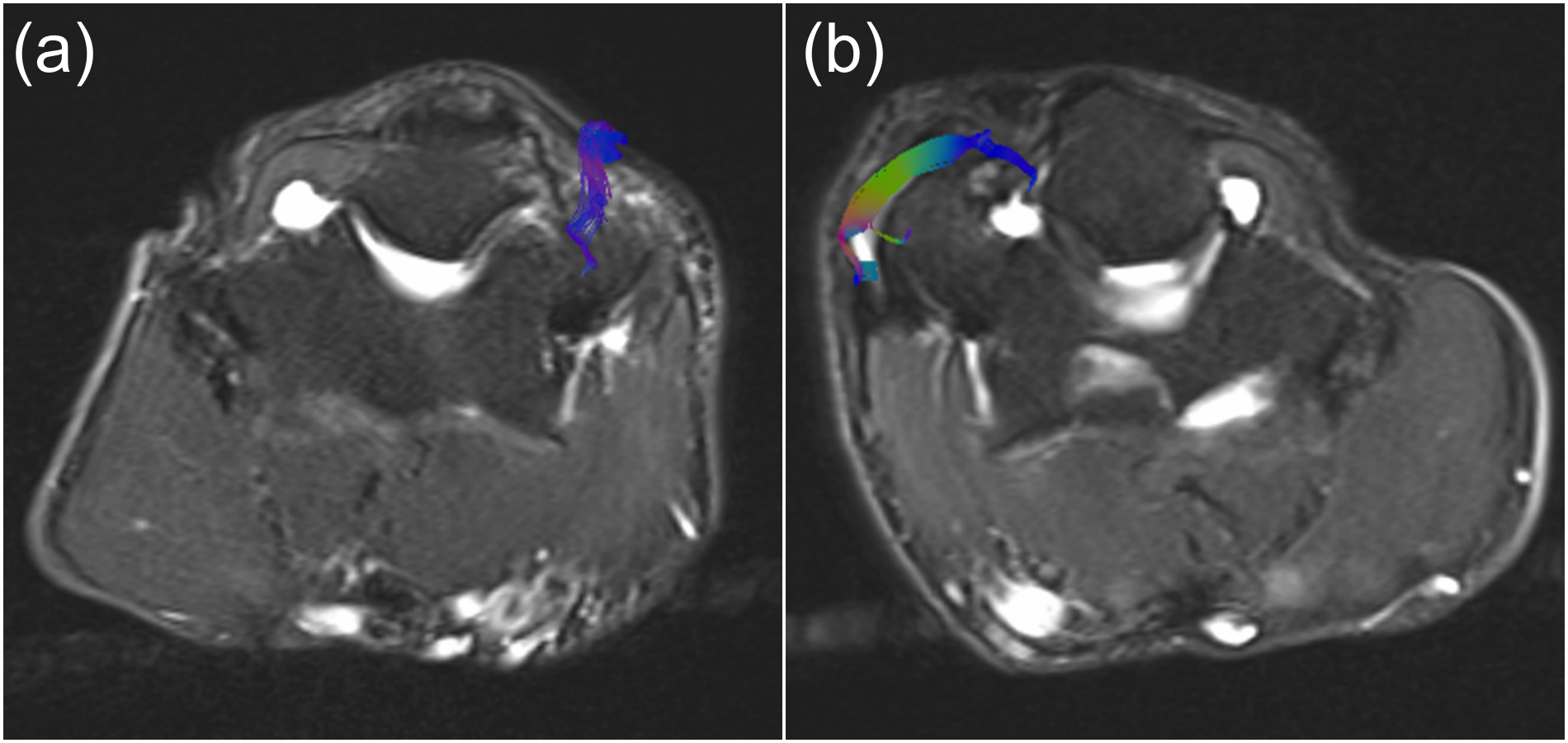

Supplement: Supplementary file 1 — Supplementary Fig. 1. DTI fiber tractography reconstructions of the right and left ulnar nerves overlaid with axial/transverse morphological MRI sequences. a On the right, the ulnar nerve runs posterior to the medial epicondyle within the cubital tunnel, b while on the left the nerve is abnormally located anterior to the medial epicondyle. (TIFF 6680 KB) [file 276_2021_2881_MOESM1_ESM.tiff]
